# Supplementary material for: Prediction of inter-chain distance maps of protein complexes with 2D attention-based deep neural networks
Source: Nat Commun. 2022 Nov 15;13:6963. doi: 10.1038/s41467-022-34600-2 (PMC9666547; doi:10.1038/s41467-022-34600-2)
Supplement: Supplementary file 1 — Supplementary Info File #1 [file 41467_2022_34600_MOESM1_ESM.pdf]

Supplementary Information

I. Supplementary Tables

Supplementary Table 1. The number of sequences (N) and the number of effective sequences (Neff) of two kinds of MSAs for each homodimer in Homotest1 as well as the top L/2 inter-chain contact precision of four methods. CDPred_BFD use BFD MSAs as input; CDPred_Uniclsut use Uniclust database MSAs as input; CDPred_ComMSA uses the combination of BFD MSAs and Uniclust MSAs as input; and CDPred uses the average of the inter-chain contact map from CDPred_BFD and CDPred_Uniclust as the final prediction. The average top L/5 precision on the dataset is reported on the last row.

HomoTest1	BFD MSAs		Uniclust MSAs		CDPred_ BFD	CDPred_ Uniclust	CDPred_ ComMSA	CDPred
**Target ids**	**N**	**Neff**	**N**	**Neff**	**top L/2**	**top L/2**	**top L/2**	**top L/2**
T0759A_T0759B	847.00	270.54	493.00	109.36	0.00	2.13	0.00	0.00
T0764A_T0764B	2722.00	1407.37	60020.00	2263.96	98.08	97.44	97.44	98.72
T0770A_T0770B	3874.00	3292.85	17395.00	3715.04	6.42	10.09	15.60	6.88
T0776A_T0776B	3316.00	2796.17	41198.00	13430.78	75.47	68.87	85.85	68.87
T0780A_T0780B	2661.00	2461.21	4118.00	1915.76	0.00	0.00	0.00	0.00
T0792A_T0792B	45.00	10.41	55.00	13.49	78.38	59.46	91.89	67.57
T0801A_T0801B	3156.00	2492.77	55451.00	9025.92	31.61	39.66	41.38	47.13
T0805A_T0805B	2682.00	2390.03	20683.00	6579.58	100.00	100.00	100.00	100.00
T0811A_T0811B	2702.00	2270.96	4578.00	1724.65	99.20	97.60	99.20	99.20
T0813A_T0813B	3827.00	2421.90	27445.00	1968.25	100.00	98.61	99.31	99.31
T0815A_T0815B	2725.00	2412.33	32911.00	12048.30	11.54	0.00	0.00	3.85
T0819A_T0819B	4116.00	1225.98	53618.00	11813.40	59.78	77.65	66.48	71.51
T0825A_T0825B	3126.00	3007.67	53785.00	19819.61	0.00	0.00	0.00	0.00
T0843A_T0843B	3858.00	1947.82	30072.00	7662.40	79.35	95.65	93.48	91.30
T0847A_T0847B	2389.00	1968.35	24963.00	6526.43	84.34	62.65	97.59	84.34
T0849A_T0849B	2672.00	2135.94	37250.00	8138.64	95.37	95.37	95.37	95.37
T0851A_T0851B	1370.00	121.83	48115.00	1910.74	84.93	90.87	84.93	89.50
T0852A_T0852B	2873.00	734.33	25025.00	667.27	67.82	65.52	67.82	66.09
T0893A_T0893B	3411.00	1922.28	58033.00	17718.90	68.60	76.03	76.03	77.69
T0965A_T0965B	3584.00	2248.38	47628.00	13492.21	26.80	75.16	27.45	54.90
T0966A_T0966B	14.00	9.00	422.00	17.58	0.00	0.42	0.00	0.00
T0976A_T0976B	808.00	154.50	26844.00	1389.13	0.00	2.46	0.00	0.82
T0984A_T0984B	1413.00	313.09	22259.00	1252.01	1.94	1.94	17.10	1.94
T0999D1A_T0999D1B	3843.00	488.18	14050.00	2382.35	61.38	40.74	64.02	66.67
T0999D4A_T0999D4B	2558.00	2225.91	2187.00	1064.10	70.59	75.63	71.43	73.11
T1003A_T1003B	3991.00	1931.02	34010.00	4843.16	83.33	90.00	93.33	86.67
T1006A_T1006B	2338.00	2205.08	11717.00	4336.99	2.70	0.00	0.00	0.00
T1032A_T1032B	2122.00	1960.42	7258.00	2227.52	90.36	91.57	57.83	93.98
**Average**					**52.78**	**54.13**	**55.13**	**55.19**

Supplementary Table 2. The number of sequences (N) and the number of effective sequences (Neff) of two kinds of MSAs for each homodimer in Homotest1 as well as the top L/2 inter-chain contact precision of four methods. The average top L/5 precision on the dataset is reported on the last row.

**HomoTest2**	**BFD MSAs**		**Uniclust MSAs**		**CDPred BFD**	**CDPred Uniclust**	**CDPred ComMSA**	**CDPred**
**Target ids**	**N**	**Neff**	**N**	**Neff**	**top L/2**	**top L/2**	**top L/2**	**top L/2**
T0965A_T0965B	3584.00	2248.38	47628.00	13492.21	26.14	78.43	28.10	54.25
T0966A_T0966B	14.00	9.00	422.00	17.58	0.00	0.00	0.00	0.00
T0970A_T0970B	240.00	45.77	367.00	29.11	4.44	6.67	8.89	13.33
T0973A_T0973B	80.00	41.37	40.00	28.33	96.88	96.88	100.00	95.31
T0976A_T0976B	808.00	154.50	26844.00	1389.13	4.10	1.64	4.10	3.28
T0983A_T0983B	3640.00	1171.37	54727.00	2555.44	14.91	0.88	6.14	10.53
T0991A_T0991B	1.00	1.00	1.00	1.00	40.00	40.00	40.00	56.36
T0997A_T0997B	2530.00	939.87	3663.00	260.84	0.00	0.00	4.35	0.00
T0998A_T0998B	1.00	1.00	3.00	3.00	0.00	53.01	53.01	1.20
T1000A_T1000B	3413.00	845.48	1776.00	292.46	56.62	55.25	5.94	60.27
T1001A_T1001B	90.00	59.63	6.00	2.42	1.45	11.59	1.45	17.39
T1003A_T1003B	3991.00	1931.02	34010.00	4843.16	85.24	89.05	91.43	88.10
T1006A_T1006B	2338.00	2205.08	11717.00	4336.99	2.70	0.00	0.00	0.00
T1010A_T1010B	2626.00	330.11	15.00	6.00	38.10	6.67	38.10	16.19
T1016A_T1016B	2469.00	2088.39	31573.00	9134.52	4.95	93.07	24.75	64.36
T1018A_T1018B	3592.00	1102.84	21116.00	2595.55	7.78	15.57	13.77	15.57
T1032A_T1032B	2122.00	1960.42	7258.00	2227.52	92.77	95.18	62.65	95.18
T1038A_T1038B	23.00	6.59	26.00	6.20	23.40	31.91	26.60	24.47
T1054A_T1054B	3116.00	1824.15	5798.00	1358.38	29.58	12.68	21.13	12.68
T1078A_T1078B	218.00	88.71	241.00	90.92	64.52	45.16	48.39	61.29
T1083A_T1083B	2097.00	1041.43	241.00	69.20	70.21	21.28	80.85	65.96
T1084A_T1084B	943.00	108.68	49.00	22.73	94.29	88.57	94.29	94.29
T1087A_T1087B	2157.00	419.30	2.00	2.00	68.18	18.18	77.27	27.27
**Average**					**35.92**	**37.46**	**36.14**	**38.14**

Supplementary Table 3. The evaluation of contact predictions on the HeteroTest1 test dataset for the CDPred(A_B), CDPred(B_A), and CDPred. Ls: the sequence length of the shorter monomer in a heterodimer. Bold numbers denote the best metrics.

	top 5	top 10	top Ls/10	top Ls/5	top Ls/2	top Ls	AccOrder(‰)	AccRate Top10	AUC
CDPred(A_B)	48.89	47.78	48.83	40.92	35.71	30.13	**13.55**	**77.78**	**0.82**
CDPred(B_A)	51.11	50.00	50.03	46.52	37.72	32.56	22.32	66.67	**0.82**
CDPred	**55.56**	**54.44**	**51.47**	**47.59**	**38.64**	**32.73**	16.90	**77.78**	0.81

Supplementary Table 4. The evaluation of contact predictions on the HeteroTest2 test dataset for the CDPred(A_B), CDPred(B_A), and CDPred. Ls: the sequence length of the shorter monomer in a heterodimer. Bold numbers denote the best metrics.

	top 5	top 10	top Ls/10	top Ls/5	top Ls/2	top Ls	AccOrder(‰)	AccRate Top10	AUC
CDPred(A_B)	**24.73**	**24.55**	**24.59**	**24.22**	20.25	17.51	**40.98**	**32.73**	**0.79**
CDPred(B_A)	23.27	23.45	22.49	22.13	**20.40**	**17.53**	64.70	29.09	0.74
CDPred	23.27	23.82	23.93	22.87	20.17	17.51	62.14	**32.73**	0.77

Supplementary Table 5. The TM-score of the AlphaFold predicted tertiary structure for the monomer unit of each homodimer in HomoTest1 dataset and the corresponding top L/5, topL/2, and top L (L is the length of the monomer sequence) inter-chain contact prediction precision of CDPred.

	**Monomer TM-score**	**top L/5**	**top L/2**	**top L**
T0759A_T0759B	0.78	0.00	0.00	0.00
T0764A_T0764B	0.99	100.00	98.72	93.93
T0770A_T0770B	0.97	13.79	6.88	4.36
T0776A_T0776B	0.98	80.95	68.87	55.19
T0780A_T0780B	0.96	0.00	0.00	0.00
T0792A_T0792B	0.96	73.33	67.57	64.00
T0801A_T0801B	0.96	59.42	47.13	34.77
T0805A_T0805B	0.96	100.00	100.00	99.49
T0811A_T0811B	0.99	100.00	99.20	95.62
T0813A_T0813B	0.98	100.00	99.31	95.49
T0815A_T0815B	0.96	0.00	3.85	1.92
T0819A_T0819B	0.97	85.92	71.51	69.08
T0825A_T0825B	0.97	0.00	0.00	0.00
T0843A_T0843B	0.99	100.00	91.30	82.88
T0847A_T0847B	0.99	90.91	84.34	70.48
T0849A_T0849B	0.97	100.00	95.37	87.56
T0851A_T0851B	0.98	96.55	89.50	78.82
T0852A_T0852B	0.93	79.71	66.09	54.02
T0893A_T0893B	0.71	87.50	77.69	56.20
T0965A_T0965B	0.99	77.05	54.90	45.75
T0966A_T0966B	0.95	0.00	0.00	0.00
T0976A_T0976B	0.98	0.00	0.82	1.23
T0984A_T0984B	0.98	2.42	1.94	1.29
T0999D1A_T0999D1B	0.97	77.33	66.67	50.53
T0999D4A_T0999D4B	0.98	85.11	73.11	56.72
T1003A_T1003B	1.00	96.43	86.67	79.10
T1006A_T1006B	0.99	0.00	0.00	5.41
T1032A_T1032B	0.70	100.00	93.98	88.55

**Average**	**0.95**	**60.94**	**55.19**	**49.01**

Supplementary Table 6. The TM-score of the AlphaFold predicted tertiary structure for the monomer unit of each homodimer in HomoTest2 dataset and the corresponding top L/5, top L/2, and top L (L is the length of the monomer sequence) inter-chain contact prediction precision of CDPred.

	**Monomer TM-score**	**top L/5**	**top L/2**	**top L**
T0965A_T0965B	0.99	80.33	54.25	45.42
T0966A_T0966B	0.95	0.00	0.00	0.00
T0970A_T0970B	0.78	11.11	13.33	12.09
T0973A_T0973B	0.94	100.00	95.31	95.31
T0976A_T0976B	0.98	0.00	3.28	2.87
T0983A_T0983B	0.95	4.44	10.53	10.48
T0991A_T0991B	0.31	68.18	56.36	46.85
T0997A_T0997B	0.99	0.00	0.00	0.00
T0998A_T0998B	0.65	0.00	1.20	1.20
T1000A_T1000B	0.97	68.97	60.27	49.43
T1001A_T1001B	0.96	29.63	17.39	10.79
T1003A_T1003B	1.00	96.43	88.10	80.52
T1006A_T1006B	0.99	0.00	0.00	5.41
T1010A_T1010B	0.95	2.38	16.19	19.91
T1016A_T1016B	0.99	70.00	64.36	51.72
T1018A_T1018B	1.00	9.09	15.57	14.67
T1032A_T1032B	0.70	100.00	95.18	88.55
T1038A_T1038B	0.92	35.14	24.47	18.62
T1054A_T1054B	0.92	7.14	12.68	18.88
T1078A_T1078B	0.98	68.00	61.29	56.00
T1083A_T1083B	0.87	77.78	65.96	56.38
T1084A_T1084B	0.91	100.00	94.29	88.73
T1087A_T1087B	0.98	58.82	27.27	20.22
**Average**	**0.90**	**42.93**	**38.14**	**34.52**

Supplementary Table 7. The TM-score of the AlphaFold predicted tertiary structure of each of the two chain of each heterodimer in the HeteroTest1 dataset and the corresponding top Ls/5, topLs/2, and top Ls inter-chain contact prediction precision of CDPred (Ls is the length of shorter monomer sequence).

	**Chain A TM-score**	**Chain B TM-score**	**top Ls/5**	**top Ls/2**	**top Ls**
H0957A_H0957B	0.92	0.97	80.65	63.64	50.32
H0974A_H0974B	0.97	0.97	15.38	14.71	17.39
H0986A_H0986B	0.96	0.97	83.33	47.83	33.70
H1015A_H1015B	0.88	0.93	17.65	13.95	17.44
H1017A_H1017B	0.94	0.93	68.18	54.55	49.09

H1019A_H1019B	0.96	0.97	100.00	100.00	82.76
H1045A_H1045B	0.96	0.94	0.00	0.00	0.00
H1047A_H1047B	0.54	0.86	0.00	0.00	0.00
H1065A_H1065B	0.96	0.97	63.16	53.06	43.88
**Average**	**0.90**	**0.95**	**47.59**	**38.64**	**32.73**

Supplementary Table 8. The TM-score of the AlphaFold predicted tertiary structure of each of the two chains of each heterodimer in the HeteroTest2 dataset and the corresponding top Ls/5, top Ls/2, and top Ls inter-chain contact prediction precision of CDPred (Ls is the length of shorter monomer sequence).

	**Chain A TM-score**	**Chain B TM-score**	**top Ls/5**	**top Ls/2**	**top Ls**
7L2RF_7L2RB	0.50	0.95	0.00	0.00	0.00
5R4CB_5R4CC	0.98	0.98	100.00	95.74	95.79
6X1FD_6X1FE	0.98	0.80	0.00	0.00	0.00
7ALAI_7ALAA	0.99	0.68	0.00	0.00	0.00
7ALBh_7ALBI	0.98	0.97	0.00	0.00	0.00
7AOFF_7AOFG	0.94	0.97	0.00	0.00	0.00
7AP8S_7AP8E	0.52	0.95	0.00	0.00	0.00
7B8TB_7B8TE	0.97	0.99	0.00	0.00	0.00
7D3YB_7D3YD	0.60	0.58	0.00	0.00	0.00
7D7FD_7D7FA	0.95	0.85	2.25	1.35	2.02
7DBDA_7DBDB	0.96	0.98	0.00	0.00	0.00
7DNID_7DNIG	0.98	0.97	0.00	0.00	0.00
7E3BC_7E3BA	0.98	0.97	14.29	9.62	7.62
7E5OA_7E5OH	0.95	0.91	32.43	28.72	20.74
7E6GB_7E6GF	0.62	0.97	0.00	0.00	1.67
7E8EH_7E8EC	0.85	0.64	0.00	0.00	0.00
7EA6E_7EA6D	0.98	0.94	100.00	98.97	95.88
7EELF_7EELL	0.91	0.80	0.00	0.00	0.00
7EEPG_7EEPV	0.68	0.90	0.00	0.00	0.00
7EKQA_7EKQC	0.80	0.95	0.00	0.00	0.00
7EW0B_7EW0C	0.97	0.81	54.55	58.62	55.17
7F4HA_7F4HN	0.87	0.91	8.00	3.17	1.57
7F5BD_7F5BE	0.82	0.76	0.00	1.03	0.52
7FIHB_7FIHR	0.99	0.96	0.00	0.00	0.00
7JZYA_7JZYK	0.69	0.96	0.00	0.00	0.00
7K7HC_7K7HL	0.98	0.87	0.00	0.00	0.00
7KA1A_7KA1B	0.99	0.98	83.02	68.66	56.34
7KBRH_7KBRA	0.99	1.00	100.00	96.15	94.29
7KN3A_7KN3L	0.98	0.93	0.00	1.03	1.03
7L2MA_7L2ME	0.90	0.48	0.00	0.00	0.00
7L2TF_7L2TB	0.50	0.93	0.00	0.00	0.00

7LB6L_7LB6X	0.99	0.98	36.54	44.62	42.69
7LTRC_7LTRD	0.93	0.94	0.00	4.00	5.88
7LXBM_7LXBN	0.94	0.97	0.00	0.00	0.00
7LXTJ_7LXTI	0.97	0.93	60.47	50.47	33.95
7MLUC_7MLUH	0.97	0.92	0.00	0.00	0.00
7MPGG_7MPGF	0.91	0.79	88.10	88.68	77.46
7NKZB_7NKZA	0.99	0.99	91.04	85.71	74.18
7O27A_7O27B	0.97	0.83	87.18	63.64	49.75
7O28A_7O28B	0.97	0.93	79.49	64.65	52.53
7O3HE_7O3HO	0.64	0.99	0.00	0.00	0.00
7OELA_7OELB	0.96	0.93	74.36	62.63	50.00
7OI7c_7OI7h	0.98	0.93	60.00	40.00	22.00
7ONIC_7ONIH	0.84	0.86	0.00	0.00	0.00
7OOPU_7OOPV	0.48	0.21	0.00	0.00	0.00
7OZNF_7OZNG	0.97	0.95	0.00	0.00	0.00
7P5VC_7P5VI	0.90	0.97	0.00	0.00	0.00
7PDZF_7PDZI	0.95	0.96	0.00	0.00	0.00
7RCOA_7RCOD	0.81	0.90	31.82	18.18	11.71
7RKSR_7RKSL	0.97	0.72	0.00	0.00	0.00
7RS1A_7RS1B	0.97	0.80	77.27	60.91	52.27
7S0YB_7S0YA	0.95	0.99	0.00	4.44	6.11
7S0ZD_7S0ZB	0.92	0.99	0.00	0.00	1.68
7V2AF_7V2AG	0.97	0.88	77.27	58.18	50.45
7V5MF_7V5ME	0.99	0.96	0.00	0.00	0.00
**Average**	**0.89**	**0.88**	**22.87**	**20.17**	**17.51**

II. Supplementary Figures

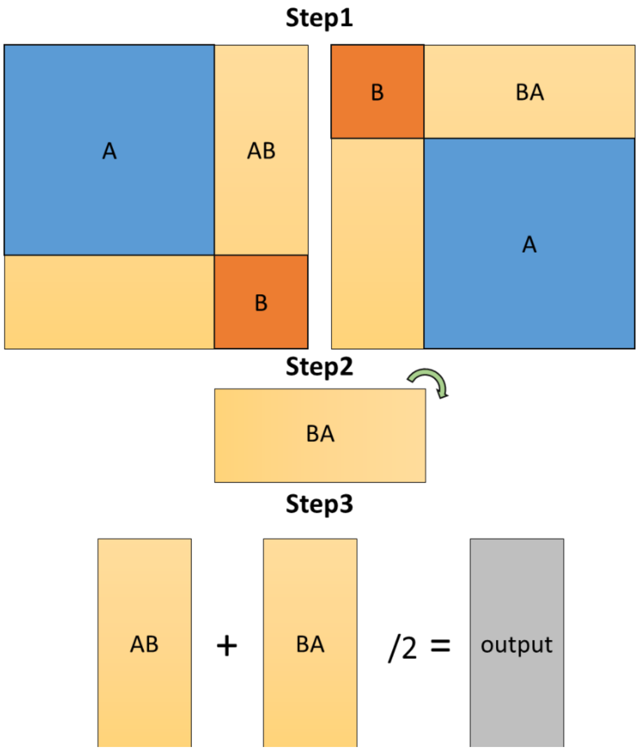

Supplementary Figure 1. The process of averaging inter-chain distance predictions from two orders of heterodimers (AB and BA) to generate the final prediction for heterodimers. Step1 is to use two orders of monomers A and B to get two predicted inter-chain contact maps. Step 2 is to take the BA inter-chain part out of the contact map predicted from the BA order and transpose it so that it shares the same shape as the AB order. Step 3 is to average AB and BA parts to generate the final inter-chain prediction result.

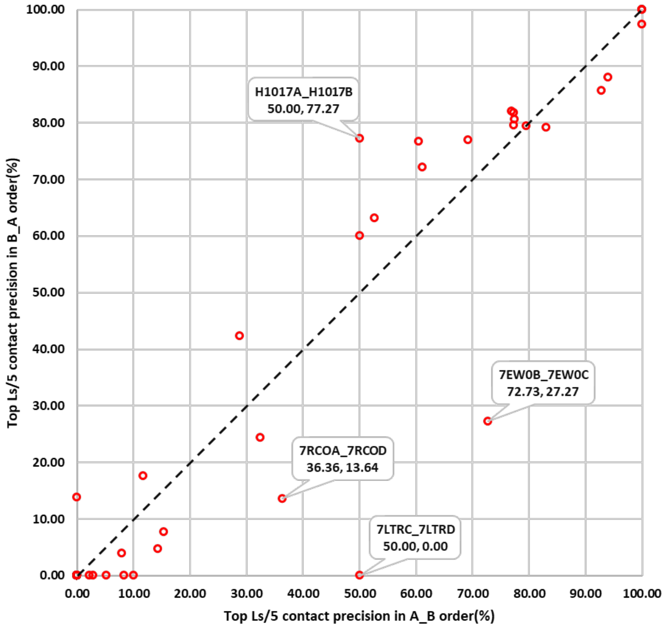

Supplementary Figure 2. Target by target comparison of top Ls/5 contact precision on HeteroTest1 and HeteroTest2 between two different orders of monomer A and monomer B (AB and BA). x-axis is the precision of the A_B order and y-axis is the precision of the B_A order. The targets with a precision difference larger than 20% for the two orders are marked in the figure.

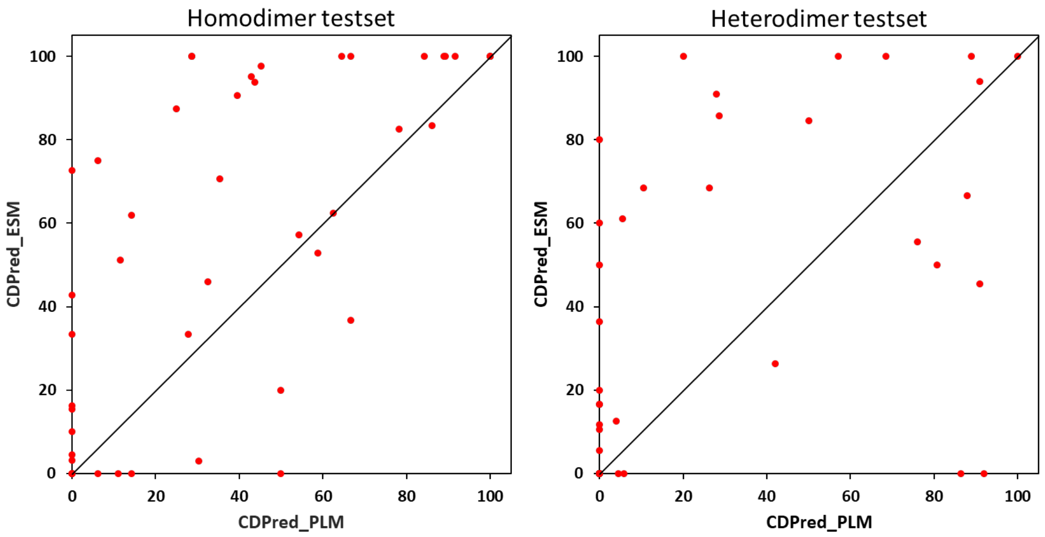

Supplementary Figure 3. The plot of the top L/10 contact prediction precision (%) of CDPred_ESM against CDPred_PLM for each dimer in the homodimer and heterodimer datasets, respectively. CDPred_ESM has higher precision (dots above the diagonal) for most dimers.

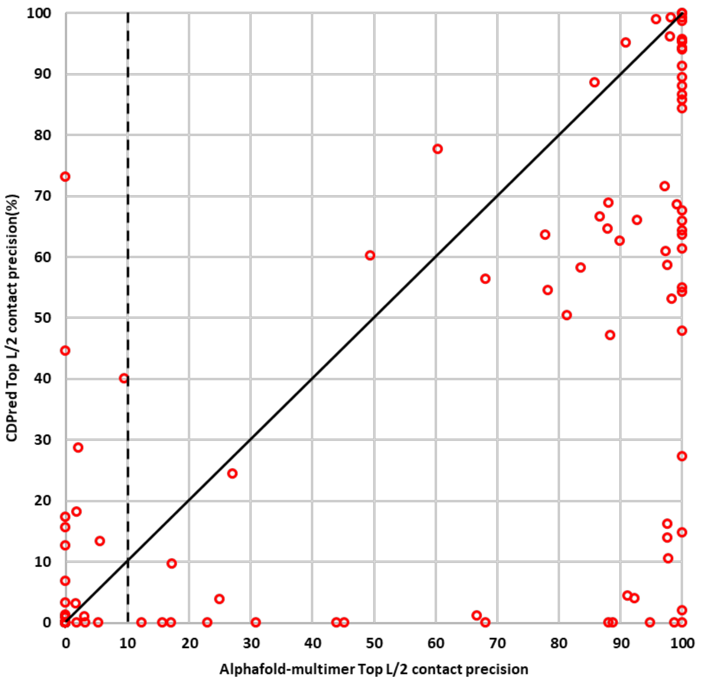

Supplementary Figure 4. The target-by-target comparison of CDPred and AlphaFold2-multimer on the four test datasets in terms of top L/2 inter-chain contact precision. When the top L/2 contact prediction precision of AlphaFold2-multimer is less than 10% (the dots on the left of the virtual vertical line), CDPred performs substantially better than AlphaFold2-multimer.
